# Symmetrical Interhemispheric Subdural Hematoma

**DOI:** 10.7759/cureus.13711

**Published:** 2021-03-05

**Authors:** Naohiro Uchio, Ryoji Miyano, Hideyuki Matsumoto

**Affiliations:** 1 Department of Neurology, Mitsui Memorial Hospital, Tokyo, JPN

**Keywords:** interhemispheric subdural hematoma, hemodialysis, falx cerebri

## Abstract

Interhemispheric subdural hematoma (ISDH) is a rare subtype of subdural hematoma. We report the case of an 81-year-old woman on hemodialysis with sudden nausea and vomiting. A computed tomography (CT) scan of the brain showed a bilaterally symmetrical increase in the thickness and density of the falx cerebri. At first, the findings were overlooked, but were later identified as an acute ISDH. The patient was treated conservatively and the symptoms completely resolved. The possibility of ISDH should be considered even if CT images of the brain are symmetrical.

## Introduction

Although subdural hematoma (SDH) is common in the elderly, interhemispheric subdural hematoma (ISDH) is a rare subtype due to the unusual location [1-5]. Here, we report the case of an initially overlooked spontaneous acute ISDH showing symmetrical axial computed tomography (CT) images of the brain. The patient’s only symptoms were headache and vomiting without neurological deficits or altered consciousness. This case has educational importance for non-specialist doctors who examine headache patients but are not familiar with the radiological findings of ISDH.

## Case presentation

**Figure 1:**
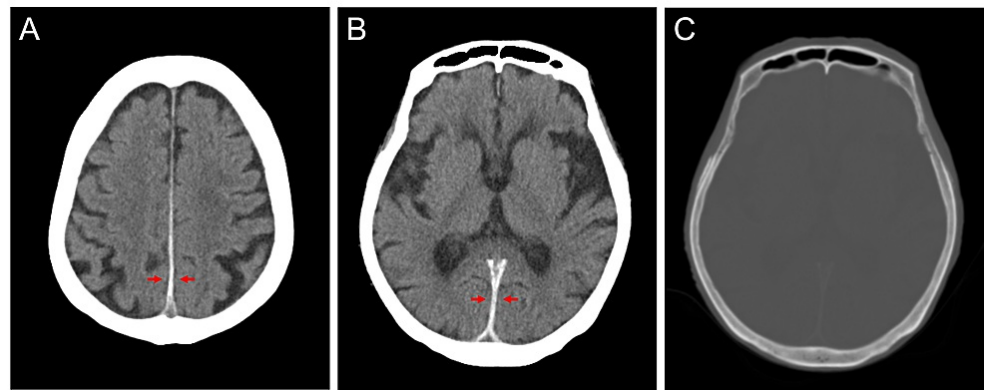
Axial images of non-contrast CT scan of the brain. CT images of the brain show a slight increase in the thickness and a high increase in the density of the posterior superior (A, arrows) and inferior (B, arrows) parts of the falx cerebri. The bone window of the same image (B) shows no calcification of the falx (C).

An 81-year-old woman with hypertension and chronic kidney disease presented with sudden nausea and vomiting. She had been on hemodialysis for four years but had never experienced these symptoms before. She had no history of trauma or seizure. Her vital signs were normal (blood pressure: 135/55 mmHg; heart rate: 86 bpm). Neurological examinations revealed no focal symptoms. Routine blood tests, including complete blood count, biochemistry, and coagulation tests, were unremarkable (white cell count: 4,900/μL; hemoglobin: 10.1 g/dL; platelet count: 186,000/μL; prothrombin time: 14.0 seconds; activated partial thromboplastin time: 36.0 seconds). Non-contrast CT of the brain showed a bilaterally symmetrical increase in the thickness and density of the falx cerebri (Figure 1). Initially, a resident physician did not notice these abnormal findings; later, however, a consultant doctor pointed out an acute ISDH based on the characteristic radiological findings. Because the hemorrhages were limited to the thin subdural spaces, the patient did not require neurosurgical intervention, and conservative therapies, including fasting and antihypertensive therapy, were performed. Her symptoms completely resolved within three days and follow-up CT scans showed no further enlargement of the SDH.

## Discussion

SDH is generally observed in the elderly following head trauma, especially if they are heavy drinkers. Hemodialysis is a known risk factor for SDH development [6], and acute SDH is seen in 9% of sudden deaths in chronic hemodialysis patients [7]. Risk factors of atraumatic spontaneous ISDH have not been sufficiently examined, but coagulopathies, aneurysmal rupture, and anticoagulation therapy have been proposed [1,4]. Our case suggests that hemodialysis may also be a risk factor for spontaneous ISDH.

The symptoms of ISDH vary from signs of intracranial hypertension, contralateral leg monoparesis or leg-dominant hemiparesis known as falx syndrome, and altered consciousness [3]. Headache is the most common symptom of ISDH [1] and, notably, headache as a sole symptom can be observed [2]. Neuroimaging surveillance would be beneficial in detecting ISDH even in headache patients without neurological deficits or altered consciousness.

In our case, the differential diagnosis of the high-density area around the falx in CT images included sinus thrombosis and dural calcification. Especially, dural calcification can be a complication of prolonged hemodialysis. However, there was no evidence to support impaired coagulation or dural calcification, suggesting that our diagnosis of ISDH was reasonable.

ISDH is mostly limited to one side of the falx because of the tight adherence of subarachnoidal trabeculations between the brain and the parasagittal dura mater, but some cases of ISDH have bilateral hematomas [3]. If the thickness of bilateral ISDH is equally thin, as shown in our case, it looks symmetrical and can be overlooked.

Regarding the treatments of ISDH, conservative therapy is preferred [1]. Surgery is useful to remove the concomitant lesions, but the ISDH itself is mostly not the target for removal [1]. The outcome of ISDH is usually benign, but its prognostic factors remain unclear. Regarding traumatic ISDH cases, severely altered consciousness, hypovolemic shock, skull fracture, convexity or posterior fossa SDH, and subarachnoid hemorrhage were correlated with poor outcomes [5]. Additional cases are needed to ascertain the factors that influence the outcome of spontaneous ISDH.

## Conclusions

Here, we described a case of initially overlooked atraumatic spontaneous acute ISDH showing symmetrical CT images of the brain. We need to consider the possibility of ISDH even if CT images of the brain appear symmetrical.
